# Physical activity, resilience, emotions, moods, and weight control of older adults during the COVID-19 global crisis

**DOI:** 10.1186/s11556-021-00258-w

**Published:** 2021-03-01

**Authors:** Sima Zach, Aviva Zeev, Miki Ophir, Sigal Eilat-Adar

**Affiliations:** The Academic College at Wingate, Wingate Institute, 4290200 Netanya, Israel

**Keywords:** Healthy behavior, COVID-19, Resilience, Depression symptoms

## Abstract

**Background:**

The main purpose of the research was to examine the differences between adults in the age group 70+ and two other age groups (45–59 and 60–69), concerning their healthy and active lifestyle. The death toll of the current COVID-19 pandemic is strongly biased toward the elderly. However, some studies of crises suggest that older adults tend to perceive events as less stressful than do younger people. Therefore, we examined healthy behavior in populations at risk according to the age cutoff-points used by the Ministry of Health at the time of vaccination, and divided the participants into three age groups (45–59, 60–69, and 70+) following health organizations’ recommendations.

**Methods:**

Participants were 1202 people, 381 males and 821 females, aged 45–90. A survey comprised of six parts was used: Demographic background, the International Physical Activity Questionnaire–short version, Positive and Negative Affect Schedule – PANAS, the Connor and Davidson Resilience Scale, a questionnaire for measuring depressive moods, and questions regarding weight change, based on the Israeli National Health and Nutrition (MABAT) survey. Data were collected in Israel during the first complete lockdown. The questionnaire was distributed via e-mail, WhatsApp, Twitter, and Facebook using a snowball sampling method.

**Results:**

Resilience and negative feelings and depression symptoms were higher in age group 45–59 compared to 70+ year-old participants, and the depression symptoms score was also higher among participants aged 45–59 compared to ages 60–69. Physical activity was associated with higher resilience, fewer depression symptoms, and fewer negative emotions. Regarding gender and psychological variables, no differences were found. During the time of lockdown, weight change was not prevalent and sleeping hours increased.

**Conclusion:**

In adults at 70+, the physical activity level, physical activity before and during the lockdown, emotions, sleeping hours, and weight change were similar to the other adult groups that were examined (45–59 and 60–69). However, in the older adults groups (70+ and 60–69), resilience and depression symptoms were lower than in the youngest age group.

## Introduction

The global outbreak of the COVID-19 plague in early 2020 has paralyzed the entire world. The sudden and rapid spread of the pandemic has shattered the normality of daily life, with a long-term impact that has yet to be assessed [1]. Governments’ response efforts have been directed at restraining the transmission of the virus, and lockdowns were imposed. Universities, colleges, and entire education systems, in addition to many work places, transitioned to online activity instead of physical presence. Businesses, public places, restaurants, and parks were shut down. Flights were suspended, airports and borders were closed. These restrictions were aimed at ensuring social distancing, limiting the movement of the population, and curtailing the effect of the pandemic. Although this strategy was reported to be effective for decelerating the COVID-19 outbreak, the subsequent quarantine was also reported as being associated with harmful implications to society [2, 3]. This unique phenomenon has never occurred/been observed before; therefore, the extent of its implications on a variety of aspects of life is still unknown.

A recent review on the psychological impact of quarantine as a result of past epidemic disease outbreaks, such as Ebola, SARS, H1N1, and equine influenza, showed that the psychological health and well-being of people who were forced to go through periods of isolation were negatively affected [4]. Following this line of research, investigators in European countries such as Italy [5], Switzerland [6], and Spain [7], in the UK [8] and the USA [9], and in countries in Asia such as China [10] and Singapore [11], sought to examine the impact of social distancing and isolation on the mental health of the population.

Imposed isolation has a huge impact on many aspects of people’s lives, causing considerable psychological strain and triggering a variety of psychological conditions [5]. People are separated from loved ones for unknown durations, they experience uncertainty concerning the disease status due to contradictory messages from health authorities, and they feel a loss of control, a sense of being trapped [12], a threat to their resilience and the well-being of their family [13], and a loss of freedom that on occasion creates dramatic effects [4]. Mass quarantine is likely to substantially raise people’s fears, since it shows that the authorities believe the situation to be severe and likely to worsen [12]. Psychological consequences that were reported include post-traumatic stress, depression symptoms, anxiety, irritability, impatience, tension, and anger [4, 12, 14]. Longer-term effects were also considered to be possible. Furthermore, quarantine might lead to physical inactivity, which contributes to adverse health changes such as cardiovascular vulnerability and decreased aerobic capacity [15]. The literature has extensively described the positive contribution of physical activity to general health [16–18]. Hence, long term inactivity, as has been forced upon us by the COVID-19 pandemic, might lead to a marked decline of health, both physical and mental.

The spotlight of the current article is related to public health. The death toll of the current COVID-19 pandemic is strongly biased toward the elderly. However, some studies of crises suggest that older adults tend to perceive events as less stressful than do younger people [19, 20]. Therefore, we examined healthy behavior in at-risk populations according to age cutoff-points used by the Ministry of Health at the time of vaccination, and divided the participants into three age groups (45–59, 60–69, and 70+) following health organizations’ recommendations [21–23].

We were seeking to identify both resilience factors and risk factors, in order to report the population’s needs in the current crisis and to assess their implications. The main purpose of the research was to examine the differences between adults, age group 70+, and another two age groups (45–59 and 60–69), concerning their healthy and active lifestyle. More specifically: (1) to determine if the participants did or did not regularly participate in physical activity before the lockdown, and whether this changed after the lockdown; (2) to examine their level of physical activity during the lockdown, (3) to examine relationships between weight and physical activity, before and during the lockdown; (4) to examine the relationships between habits and level of physical activity, and psychological well-being variables; and (5) to examine the relationships between the level of physical activity and the duration of daily sleeping hours.

## Method

### Participants

Nonprobability snowball sampling was used to recruit participants [24]. Sample size was calculated based on two-way analysis of variance (ANOVA) by age and sex using G*Power analysis program. For an effect size of 0.2, α = 0.05 and 1-β = 0.9, a sample size of 450 participants was calculated. Since the data were collected during the first lockdown, 1202 questionnaire were eligible for the current study.

Participants were 1202 people, 381 males and 821 females, aged 45–90, from all parts of the State of Israel, representing its seven main regions. Participants reported their weight and height, and body mass index (BMI) was calculated by the researchers.

### Survey

A six-part survey was used, including:

- Demographic background.
- *The International Physical Activity Questionnaire (IPAQ)* [25] – the short version relating to physical activity that was conducted during the previous week. Participants had to describe their level of physical activity, and its frequency, duration, and intensity. See the original instrument for further details.
- *Positive and Negative Affect Schedule –* PANAS [26]. A 20-item questionnaire assessed positive affects (10 items) and negative affects (10 items) experienced by the participants in the previous month. Participants rated their feelings on a 5-point scale (1 = hardly at all or not at all; 5 = to a great extent). Reliabilities, in terms of Cronbach alphas, for the original scale were .89 for positive affect and .92 for negative affect. In the current study Cronbach alphas were .83 for positive affect and .86 for negative affect.
- *The Connor and Davidson Resilience Scale* [27] *– The Connor-Davidson Resilience scale (CD-*^RIS^*C)* is comprised of 25 items, each rated on a 5-point scale (0–4), with a higher score reflecting greater resilience. Internal consistency of the original validation study was .89, and test-retest reliability demonstrated a high level of agreement between the two tests, with an intraclass correlation coefficient of .87. Factor analysis in the current study yielded two factors: (1) Personal competence and self-control, and (2) Positive acceptance of change. Internal consistency ranged from .87 to .90.
- *A questionnaire for measuring depressive moods* [28] *–* Six questions on a 4-point scale measured depressive symptoms. Internal consistency for the current sample was .86.
- Questions regarding weight change, based on the Israeli National Health and Nutrition (MABAT) survey, questions 50–54 [29].

### Procedure

Data were collected during the complete lockdown from April 14, 2020 to May 6, 2020. The survey was approved by the Institutional Review Board (IRB), permission No. 250. The questionnaire was distributed via e-mail, WhatsApp, Twitter, and Facebook using a snowball sampling method. All participants were asked to share the link of the questionnaire with others in order to obtain a wider sample.

### Data analysis

Physical activity was defined as the answer to the question: “Were you engaged in physical activity before the lockdown?”, along with the answer to the question: “Are you engaged in physical activity during the lockdown?”, and categorized into a dichotomous variable: Yes or No. Reported adherence to physical activity prior to the pandemic, and during the lockdown, was categorized into four groups: (a) No/No – have not done physical activity before the lockdown/not doing physical activity during the lockdown; (b) No/Yes – have not done physical activity before the lockdown/doing physical activity during the lockdown; (c) Yes/No – did physical activity before the lockdown/not doing physical activity during the lockdown; (d) Yes/Yes – did physical activity before the lockdown/doing physical activity during the lockdown. In addition, participants were categorized into three groups according to the American College of Sports Medicine (ACSM) recommendations for healthy and active lifestyle [30], as follows: Inactive, insufficiently active – those who are doing less than 150 min of Moderate-to-Vigorous Physical Activity (MVPA) per week, and sufficiently active – those who are doing 150+ minutes of MVPA per week. The background characteristics of the four groups of physical activity behavior are presented by means and standard deviations for normal variables, and by frequency for categorical data.

Confirmatory Factor analysis was conducted to reconfirm the behavior factors for the current cohort. The number of factors to retain was calculated using the Kaiser-Guttman rule (eigenvalue > 1) and the scree plot. Two factors were created for the resilience scale, one factor for the depression scale, and two factors for the PANAS scale with eigenvalues values > 1.

One-way ANOVA with Bonferroni correction was conducted to compare the reported psychological variables among the four groups of physical activity behavior, and a Chi Square test was used to compare weight change in those four groups, using the ALSCAL procedure in IBM SPSS (Version 25.0). Participants were divided into three age groups: 45–59, 60–69, and 70+. Chi-Square tests were conducted to examine the differences between the groups on baseline variables.

## Results

Means, SDs, and significant differences in basic variables according to three age groups are presented in Table 1.

**Table 1:** Survey variables according to age groups means (SD) for continuous variables, n (%) for categorical variables

Age (years)	45–59	60–69	70+
	*n* = 645	*n* = 393	*n* = 164
BMI (Kg/m^2^)	25.91(4.56)	26.61(4.36)(a*)	25.88(4.09)
Weight change (Kg)	.54(1.91)	.45(1.71)	.44(2.39)
Female (%)	463(71.8)	255(64.9)	103(63.2)
Male (%)	182(28.2)	138(35.1)	60(36.8)
Not-active	168(26.0)	124(19.2)	353(54.7)
Insufficiently active	89(22.6)	60(15.3)	244(62.1)
Active	49(30.1)	21(12.9)	93(57.1)
Resilience			
Positive acceptance of change	3.05(0.74)(c*)	2.97(0.73)	2.86(0.86)
Personal ability, self-competence, and self-control	3.07(0.71)(c*)	2.98(0.74)	2.88(0.82)
PANAS-positive	2.83(0.82)	2.76(0.80)	2.72(0.91)
PANAS-negative	1.95(0.77)(c***)	1.87(0.68)	1.71(0.62)
Depression symptoms	2.01(0.65)(b**,c***)	1.87(0.61)	1.73(0.63)

* = *p* < .05; ** = *p* < .01; *** = *p* < .001; a = compared with 45–59; b = compared with 60–69; c = compared with + 70

Mean BMI was highest among people aged 60–69. The mean BMI in this age group was higher than the normal BMI and is considered overweight. Average weight change was not different across ages with a very large SD. On average, reported weight did not change during the first lockdown.

Most of the participants were sufficiently active during the lockdown according to ACSM criteria, with no significant difference between age groups. There was a significant difference between age groups in both Resilience factors *–* Positive acceptance of change [*F* (2; 1146) = 5.309; *p* < .01], and Personal ability, self-competence, and self-control [*F* (2; 1146) = 4.004; *p* < .05]. Post Hoc tests with Bonferroni corrections showed that both factors were higher in age groups 45–59 compared to 70+. No differences were obtained between age groups in positive feelings (PANAS positive), whereas negative feelings (PANAS negative) [*F* (2; 1072) = 3.228; *p* < .05] and depression symptoms [*F* (2; 1146) = 10.684; *p* < .001] were higher in age group 45–59 compared to 70+ year-old participants, and the depression symptoms score was also higher among participants aged 40–59 compared to those aged 60–69.

No differences appeared between the three age groups by the three categories of physical activity level [*χ*^2^ (4) = 9.122, *p* = .058]; in addition, no gender differences appeared between age groups according to the physical activity level [*χ*^2^ (4) = 7.122, *p* = .115].

Differences among the age groups according to their physical activity habits before and during the lockdown appeared only with regard to negative feelings: PANAS negative and depression symptoms (Table 2). Two-Way ANOVA (physical activity category X age group) revealed significant differences between the four physical activity categories in PANAS Negative only in age group 45–59 [*F* (2; 1072) = 3.228; *p* < .05]. Those who were not active before and began physical activity during the lockdown had a lower score than those who used to do physical activity before and stopped during the lockdown.

**Table 2:** Means and SDs of psychological variables according to age groups and physical activity before/during the lockdown

Age	PA	Resilience		PANAS		Depression Symptoms
		PAC	PA, SC & SC	Positive	Negative	
45–59	No/No	2.95(.78)	2.96(.71)	2.79(.92)	1.88(.71)	2.07(.67)
	No/Yes	3.15(.76)	3.04(.77)	2.70(.82)	1.75(.69)	1.93(.59)
	Yes/No	3.03(.73)	3.01(.72)	2.68 (.85)	2.14(.88) b	2.22(.69) b, d
	Yes/Yes	3.07(.73)	3.12(.72)	2.91(.85)	1.93 (.88)	1.93(.69)
60–69	No/No	2.68(.90)	2.66(.79)	2.27(.1.03)	1.57(.73)	1.67(.80)
	No/Yes	2.73(.79)	2.67(.87)	2.53(.86)	1.81(.48)	1.76(.55)
	Yes/No	2.95(.64)	2.95(.67)	2.75(.72)	1.84(.72)	1.90(.61)
	Yes/Yes	3.02(.74)	3.05(.72)	2.83(.80)	1.92(.70)	1.89(.61) b
70+	No/No	2.68(.90)	2.66(.79)	2.27(1.03)	1.57(.73)	1.67(.80)
	No/Yes	2.68(.69)	2.91(.70)	2.62(.75)	1.86(.74)	1.77(.61)
	Yes/No	2.88(.83)	3.00(.73)	2.73(.95)	1.74(.55)	1.92(.69)
	Yes/Yes	2.90(.90)	2.85(.87)	2.79(.89)	1.70(.62)	1.66(.56)

*b* Compared to No/Yes, *p* < .05, *d* Compared to Yes/Yes, *p* < .001, *PA* Physical activity, *PAC* Positive acceptance of change, *PA, SC, & SC* Personal ability, self-competence, and self-control

Depression was more prevalent among ages 45–59 compared to the older ages. Differences were found in the same age group in depression symptoms [*F* (3; 1146) = 4.323; *p* < .01]. Those who stopped being active during the lockdown had a higher score compared to those who were not active before, and began physical activity during the lockdown (*p* < .05); they also had a higher depression score compared with those who were active both before and during the lockdown. In age group 60–69, those who were active both before and during the lockdown scored higher compared to those who began physical activity only during the lockdown (*p* < .001).

In addition, we sought to examine the differences between the age groups in psychological variables, according to their physical activity level during the lockdown. Among the 45–59 and 60–69 age groups, sufficiently active participants demonstrated a higher level of PANAS-Positive compared to inactive people (*p* = 0.39). In age group 45–59, participants who were not active demonstrated higher depression symptoms compared with the sufficiently active people (*p* < .001), and in age group 60–69 those who were insufficiently active had the highest depression symptoms, compared to both inactive and sufficiently active people (p < .001). In age group 60–69 the sufficiently active people scored higher in Personal ability, Self competence, and Self-control than those who were not sufficiently active (*p* = .010).

Results of the Two-Way ANOVA on average sleeping hours in the last week of the lockdown showed significant differences between age groups [*F* (2; 1168) = 6.490; *p* < .01], as well as significant interaction (Age X Physical activity) [*F* (6; 1168) = 3.132; *p* < .01]. That is, those participants from the four categories of physical activity at the younger age reported on different sleeping hours than those in the four categories of physical activity at the older age. Specifically, at the younger age, the people who were not active before, but were active during the lockdown (No/Yes), slept more than others, while the group ages 60–69 *–* those who were active both before and during the lockdown *–* slept more than others (Yes/Yes), and among the oldest group, those who were neither active before nor during the lockdown (No/No), slept more than the others.

Results regarding weight change are presented in Table 3. No significant differences were demonstrated between the groups [*χ*^2^ (6) = 7.135, *p* = .309].

**Table 3:** Differences between age groups in weight change, according to their physical activity before/during the lockdown (in percentages)

Age	Weight change	No/No ***n*** = 132	No/Yes ***n*** = 91	Yes/No ***n*** = 217	Yes/Yes ***n*** = 675
**45–59**	Lose	12.6	21.2	12.3	15.7
	No change	54	57.6	54.9	60
	Gain	33.4	21.2	32.7	24.3
**60–69**	Lose	18.2	17.2	11.3	17.4
	No change	57.6	51.7	56.5	66
	Gain	24.2	31.1	32.2	16.6
**70+**	Lose	16.7	20	21.4	15.7
	No change	58.3	50	54.8	73
	Gain	25	30	23.8	11.3

## Discussion

Three main and interesting findings appear from the data: Firstly, the youngest age group demonstrated a higher resilience compared to the older age groups. This result is in line with the findings of other surveys concerning mental health and psychological aspects during the COVID-19 period, which were conducted in China [10], the USA [9], Switzerland [6], and Spain [7], and reported that young adults demonstrated a high level of resilience compared to older adults.

One explanation for this finding lies in the compulsory requirement for social distancing, according to which the older adults should not meet people and should not be visited for fear of being infected. Thus, in addition to the accumulated epidemiological data on the fact that the disease mainly affects the elderly, which serves as a source of stress in itself, there is also the social component of isolation that probably impaired mental resilience among middle-aged and older adults. Researchers who compared the level of resilience of young adults before and during the disease [31] concluded that the changes in resilience levels were more consistently associated with young adults’ emotional distress than with COVID-19-related health risk exposures.

Secondly, an interesting and noteworthy finding is that in addition to the fact that the youngest group reported a higher level of resilience compared to older adults, they also reported higher levels of depressive symptoms compared to older adults. Hence, it can be concluded that people can feel resilience and depression at the same time. The fact that young people suffered from depression symptoms during the lockdown at a greater level than adults is attributed to a number of reasons: (1) A sense of suffocation due to the forced isolation and inability to lead a routine life [5], (2) A loss of sources of income and/or economic stability [12]. In contrast, older adults are at the end of their careers at work, or in retirement, and the economic threat is less tangible for them; (3) Social isolation from family members and friends [4, 6]. On the other hand, the adults, who were used to hosting their children and grandchildren found plenty of free time for themselves during this period, and initiated activities that probably calmed them down; (4) The prohibition on recreation, travel, shopping, and cultural consumption in any way that constitutes quality utilization of leisure time [2].

A possible explanation for the combination of high mental resilience and symptoms of high-level depression is that this pattern may characterize people who are distressed but must function. Both are due to the fact that the duration of the period of distress is unknown and is expected to continue, and to the fact that they have families with children and must take care of their well-being in every way possible, despite of the difficulties they are experiencing [13].

Since most of the participants were sufficiently active according to ACSM criteria during the lockdown, without a significant difference between age groups, we compared the ages in the psychological variables, according to their habits of doing activity before and during lockdown.

In other words, although we hypothesized that the activity habits of the different age groups would be reflected in the psychological metrics differently, we did not find any evidence to suggest this, except among the younger adults (ages 45–59). Higher negative emotions were reported among those who used to exercise before the quarantine and stopped during the quarantine, compared to those who did not exercise before the quarantine and started exercising during the quarantine. This finding can be explained with reference to physical activity. Others have extensively reported the relationship between physical activity and positive feelings [32–34], and vice versa. Being inactive, especially among people who used to be active and unwillingly had to change this habit, reported negative emotions including frustration, anger, despair, and depression [35, 36].

Similarly, among the youngest age group it was found that people who stopped exercising during the lockdown reported higher depressive symptoms compared to those who were and continued to be active, as well as compared to those who were inactive and started exercising during quarantine. This finding reinforces the knowledge accumulated in the research literature on the relationship between physical activity and depression in routine times (see reviews [37, 38]), in crisis/emergency time in general [39], and in COVID-19 time in particular [40]. In addition, people aged 60–69 who did and continued to do physical activity reported higher levels of depression compared to those who did not exercise and started doing physical activity during the lockdown period. That is to say, when people with a sedentary lifestyle change their habits and begin performing physical activity, they gain immediate benefits, both physically and psychologically, such as in the sense of well-being [41], happiness [42], satisfaction of life [43, 44], and a decrease in negative emotions [45].

Two additional aspects that were examined in the current research were sleeping hours and weight change. No differences were found in weight change between the age groups according to physical activity habits. This result is different from studies that were conducted in India [46] and in the USA [47], which reported that people who went through lockdown during the time of the COVID-19 disease gained weight. However, these studies were conducted on small sample sizes (a few dozen participants). In contrast, the current study was conducted with a large number of participants, and the findings obtained are similar to those reported in a study conducted in Spain in the same period on a sample of 4379 participants aged 16–84, in which most participants maintained their weight during the quarantine period [48]. This finding may be due to the fact that the first lockdown lasted a relatively short period of time. It is possible that staying in quarantine for a longer period of time would have revealed a different picture regarding weight changes in general and in regard to physical activity habits in particular.

Differences were found between age groups according to their physical activity habits and in their sleep duration per night. Among the younger participants, a positive relationship was found between physical activity and the number of sleeping hours, while in the older ones a positive relationship was found between inactivity and the duration of sleep. It would be interesting to examine in further research the relationship between the type and intensity of physical activity and the duration and quality of sleeping hours at different ages.

The current study has some limitations: The sample does not represent the entire Israeli adult population, but those who have access to computers and use them. Also, this is a self-administered questionnaire and therefore a recall bias may occur. Still, due to the fact that it is a large sample, it probably indicates/expresses a typical mood.

## Conclusions

In adults at 70+, physical activity level, physical activity before and during the lockdown, emotions, sleeping hours, and weight change were similar to other adult groups that were examined (45–59 and 60–69). However, in the older adults’ groups (70+ and 60–69), resilience and depression symptoms were lower than in the youngest age group.

## Data Availability

The datasets generated and/or analysed during the current study are not publicly available due to institutional restrictions but are available from the corresponding author on reasonable request.

## Abbreviations

PA: Physical activity
PAC: Positive acceptance of change
PA, SC, & SC: Personal ability, self-competence, and self-control
ACSM: American College of Sports Medicine
BMI: Body mass index
IPAQ: The International Physical Activity Questionnaire
PANAS: Positive and Negative Affect Schedule
